# CINet: A Constraint- and Interaction-Based Network for Remote Sensing Change Detection

**DOI:** 10.3390/s25010103

**Published:** 2024-12-27

**Authors:** Geng Wei, Bingxian Shi, Cheng Wang, Junbo Wang, Xiaolin Zhu

**Affiliations:** 1 School of Physics and Electronics, Nanning Normal University, Nanning 530100, China; shi_bingxian@163.com (B.S.); wangjunbo0313@163.com (J.W.); zxlxiaolin2020@163.com (X.Z.); 2 School of Electronic Information and Communications, Huazhong University of Science and Technology, Wuhan 430074, China; wangchust@hust.edu.cn

**Keywords:** remote sensing change detection, deep learning, constraint, interaction

## Abstract

Remote sensing change detection (RSCD), which utilizes dual-temporal images to predict change locations, plays an essential role in long-term Earth observation missions. Although many deep learning based RSCD models perform well, challenges remain in effectively extracting change information between dual-temporal images and fully leveraging interactions between their feature maps. To address these challenges, a constraint- and interaction-based network (CINet) for RSCD is proposed. Firstly, a constraint mechanism is introduced that uses labels to control the backbone of the network during training to enhance the consistency of the unchanged regions and the differences between the changed regions in the extracted dual-temporal images. Secondly, a Cross-Spatial-Channel Attention (CSCA) module is proposed, which realizes the interaction of valid information between dual-temporal feature maps through channels and spatial attention and uses multi-level information for more accurate detection. The verification results show that compared with advanced parallel methods, CINet achieved the highest F1 scores on all six widely used remote sensing benchmark datasets, reaching a maximum of 92.00 (on LEVIR-CD dataset). These results highlight the excellent ability of CINet to detect changes in various practical scenarios, demonstrating the effectiveness and feasibility of the proposed constraint enhancement and CSCA module.

## 1. Introduction

**Figure 1:**
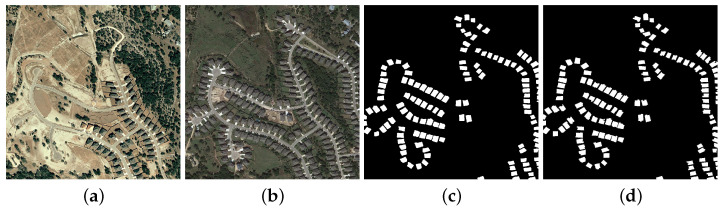
The RSCD task on LEVIR-CD. (**a**,**b**) Input remote sensing images, (**c**) ground truth, and (**d**) prediction result of CINet.

The main task of remote sensing change detection (RSCD) is to identify changes in the Earth’s surface using multi-temporal aerial and satellite imagery, which needs to analyze the acquired images and generate the resulting plots to clearly show whether the monitored area has changed, as seen in Figure 1. In recent years, RSCD technology has been widely applied in many fields such as urban planning, environmental monitoring, and resource management.

However, there are many challenges [1,2,3] associated with RSCD. This is due to the different weather, light, and spatial positions when taking remote sensing multi-temporal images, which makes the image quality taken at different times uneven. Noise and artifacts also contribute to this problem. Image registration errors can further complicate analysis. Variations in lighting, shadows, and angles of view add another level of difficulty, along with the complex nature of dynamic landscapes. Additionally, issues such as scale and spatial diversity of scenes create additional challenges. When shooting a real-world changing scene, the size of the changed and unchanged areas is not consistent, which makes it more difficult to detect changes. Recovering tagged data can be difficult, and time synchronization problems can occur. Finally, it is necessary to have models that demonstrate effective generalization capabilities. Therefore, it is crucial to design a network with strong identification capabilities to solve these problems.

Change detection compares two phasic images taken at different times, represented as $X_{1}$ and $X_{2}$, to identify and analyze the changes that occur between them. It typically involves comparing pixel-level differences between the dual-temporal images and relating these differences to a specific target or outcome, represented as Y. This comparison process can be formalized by using the techniques such as minimizing a loss function, denoted as $\min_{\theta }L\left(F_{\theta }\left(X_{1},X_{2}\right),Y\right)$, where L is the accuracy of change detection, $F_{\theta }$ is the detection model, and θ represents the parameters of the detection model. Therefore, it is essential to make L as small as possible by using reasonable methods for handling $X_{1}$ and $X_{2}$.

With the rapid development of deep learning technology, many deep learning-based network architectures have been proposed and applied to RSCD tasks. The main contribution of these methods is that they can automatically learn complex feature representations, effectively extract spatiotemporal features from large amounts of remote sensing data, and thereby improve the accuracy and efficiency of change detection. For example, Fully Convolutional Early Fusion (FC-EF) [4] used a fully convolutional positive value learning machine combined with feature representation for change detection. The fully convolutional Siamese architecture using skip connections was first proposed, and most subsequent related networks have been improved based on this foundation. STANet [5] proposed an attention module for obtaining spatiotemporal dependencies at multiple scales. It is capable of automatically learning spatiotemporal features, effectively enhancing the perception of dynamically changing areas in the image. The main contribution of ChangeFormer [6] is to introduce the transformer model into change detection tasks. It provides a new approach to the spatial and temporal complexity in change detection, applying the advantages of transformers to remote sensing image change detection. Bitemporal Image Transformer(BiT) [7] effectively extracts and models features of dual-temporal images by introducing a converter architecture and self attention mechanism in deep learning. It also improves detection accuracy through pre-training and fine-tuning mechanisms, providing a more effective solution for remote sensing change detection. MetaChanger [8] explored the importance of interaction in RSCD networks and achieved high detection performance through simple random exchanges (channel exchange and spatial exchange). These networks can extract more valuable information from images, which is significantly superior to traditional methods. However, these methods still face two challenges in detecting change regions: (1) unstable feature extraction of change regions from dual time feature maps and (2) the existing methods cannot make reasonable use of the interaction between dual-time feature maps, resulting in irrelevant changes and adverse effects on the detection results.

To solve the previously mentioned challenges, this paper proposes an innovative network that is significantly different from existing technologies. This network, named CINet, introduces the constraint mechanisms in the network’s backbone and employs the Cross-Spatial-Channel Attention (CSCA) interaction module. The constraint mechanism is introduced into the network’s backbone, which makes the difference of extracted dual-temporal feature maps in changing regions more obvious. The CSCA module is used for efficient interaction between dual-temporal features, focusing on extracting important parts from two images and facilitating mutual attention to these critical regions. Combining these two methods can effectively improve the results of change detection. Moreover, we assessed the generalization capability of CINet using six publicly available RSCD datasets.

The contributions of this work can be summarized as follows:

(1) A new constraint mechanism is proposed for the RSCD task, which is integrated into the backbone to enhance the change detection ability. This approach provides new insights and methods for improving the performance and effectiveness of networks.

(2) Design of a Cross-Spatial-Channel Attention (CSCA) module for effective interaction between the dual-temporal feature maps. The module enhances detection performance through efficient information exchange.

(3) Extensive experimental and ablation studies are conducted on six RSCD datasets.

The rest of the paper can be organized as below. Section 2 describes previous works on change detection methods, feature interaction methods, and model constraints. Section 3 details the structure of CINet and its partial functions. Section 4 reports the experimental results and ablation studies. Finally, Section 5 gives a conclusion.

## 2. Related Work

### 2.1. Classical Change Detection Methods

Previous studies have used traditional machine learning methods for change detection tasks. These include support vector machines [9], random forests [10], principal component analysis [11], and change vector analysis [12]. However, there are many problems with these traditional machine learning methods. The processing of large-scale data requires a large number of human markers, and noise interference also increases the difficulty of recognition. Moreover, these methods usually cannot make full use of timing information, resulting in low detection accuracy. This is caused by changes in weather, changes in brightness, changes in appearance, and the presence of unrelated objects. Additionally, detecting changes in different shapes and sizes is also a challenging task.

Many deep learning methods, including convolutional neural networks (CNNs) and transformers, have been used in RSCD and surpass traditional techniques [13,14,15,16]. Daudt et al. [4] introduce three key RSCD models: Among them, FC-EF uses full convolution for effective feature extraction. For the extracted feature maps, the strategy of initial fusion is adopted to effectively integrate information from multiple sources in the initial stage. Different from it, FC-Siam-Conc and FC-Siam-Diff adopt late fusion strategies, which combine the features of the middle layer, but the specific fusion methods are different. In addition to CNN-based architectures, several transformer-based models have been created that exhibit impressive performance in change detection tasks. For instance, the BiT model [7] merges CNN and transformer architectures, achieving an efficient change detection solution while maintaining a minimal parameter count. ChangeFormer [6], a pure transformer model, and the SegFormer [17] Siamese variant are designed for network depth fine-tuning to enhance performance in complex detection scenarios. They are fine-tuned for network depth to enhance performance in intricate change detection scenarios. These models are specifically fine-tuned for network depth, optimizing their performance in complex change detection scenarios. MetaChanger [8] pays special attention to the feature interaction between dual-phase images. In the feature extraction stage, the interaction is realized by simple random interchange, which verifies that the interaction is very necessary in the change detection task. In summary, significant progress has been made in the field of the RSCD, highlighting that deep learning methods have very high potential in this field. Different from the existing research, we introduce a novel network named CINet by introducing the constraint mechanisms in the network’s backbone and employing the Cross-Spatial-Channel Attention (CSCA) interaction module to interact with the effective feature information between dual-temporal feature maps.

### 2.2. Feature Interaction

Feature interaction technology plays an important role in deep learning, enhancing the expressive power of models through different interaction mechanisms, which has been widely used in tasks such as image classification [18], object detection [19], and natural language processing [20]. A common method is direct fusion, which involves simple addition, subtraction, and cascading. Although this method has high computational efficiency, it often fails to fully utilize the complex relationships between features, which may lead to a decrease in model performance. Another common feature interaction method is random swapping, especially in the MetaChanger [8] method, where channel and space features are randomly swapped in a dual-temporal feature map by setting random numbers. Although these methods are easy to implement, there are issues of instability and poor interpretability. In contrast, the attention mechanism provides a more refined and flexible way of feature interaction. By weighting spatial dimension features, spatial attention can focus on the importance of different regions in an image, making it particularly suitable for tasks such as object detection and semantic segmentation. Although spatial attention is effective in many cases, it is susceptible to the influence of the spatial structure of the input image and has poor robustness to complex transformations such as image rotation and scaling. Channel attention weights the features of different channels, enabling the network to adaptively focus on useful channels. For example, in convolutional networks, some channels may focus on features such as edges, textures, or colors. SENet [21] optimizes the performance of segmentation tasks by increasing the weight of important channels but also ignores some of the synergies between different channels. Common attention and cross-attention help effectively integrate information from multiple sources, capturing richer semantic information by focusing on the interrelationships between different features. They are commonly used for cross-modal tasks such as RGB-D [22] image fusion and visual question answering [23]. However, when there are significant differences between different modalities (such as semantic differences between images and text), they often find it difficult to effectively capture cross-modal correlations. In some studies, different forms of attention mechanisms have been combined together, such as DANet [24], which introduces a dual-attention mechanism (spatial and channel attention) for scene segmentation, significantly improving segmentation performance by capturing long-term dependencies between the two attention mechanisms. There is also the MixFormer [25] method, which performs bidirectional interaction between the self-attention and depth-wise separable convolution, providing complementary clues in both channel and spatial dimensions. Despite the success of these methods, there are still challenges in capturing the interactions between spatial, channel, and temporal features, especially when dealing with dynamic changes such as spatiotemporal feature interactions or change detection tasks.

Through a comprehensive review of RSCD methods in recent years [26], it can be seen that RSCD faces many challenges. We need a more refined feature interaction method to capture subtle changes between images. This requires enhancing spatial, channel, and temporal attention. The CSCA module proposed in this paper combines spatial and channel attention while optimizing temporal features. By integrating temporal context information and change features into attention computation through cross-attention, the CSCA module improves the capture of subtle changes, especially in multi-temporal change detection tasks.

### 2.3. Model Constraint

In the process of pursuing the enhanced expression ability of deep learning models, researchers are increasingly turning their attention to the effective implementation of constraints on models. These constraints are designed to improve generalization and stability, ultimately addressing common challenges such as over-fitting and interpretability. Previous research has focused on applying various constraints to model parameters to enhance both the generalization capability and stability of the models. For example, extensive studies on L1 and L2 norm constraints [27] have shown strong effectiveness in practice for reducing overfitting and encouraging sparsity. Some studies focus on imposing constraints on the output of the model to ensure that the output conforms to a specific real-world application scenario. In classification tasks, non-negative and probabilistic constraints [28] are often used to ensure the rationality and interpretability of the output. Structural constraints play a key role in deep learning model design, and these constraints can guide the topology and connectivity of the model. For example, in a convolution neural network, how the convolution and pooled layers are connected can be specified by topological constraints [29] to optimize the learning and reasoning process of the model. Recent research has increasingly focused on the application of dynamic constraints that adjust to the changes in the data or tasks. Dynamic constraint [30] techniques can help the model maintain good performance under different data distributions and environmental conditions. Despite significant progress in the application of constraints in deep learning, there are still some challenges. For example, how to effectively combine multiple constraints for optimal performance is still an open question. In addition, how to adjust the constraints to adapt to complex practical application scenarios without losing the learning ability of the model faces significant challenges.

## 3. Materials and Methods

We first describe Flow Dual-Alignment Fusion (FDAF) and the MIX-FFN, as well as the related modules used in Section 3.1. Then, in Section 3.2, we detail the overall architecture and functionality of CINet. We then cover the details of the Cross-Spatial-Channel Attention module (CSCA) in Section 3.3. Finally, the complete implementation of change constraint is elaborated in Section 3.4.

### 3.1. Notation and Preliminaries

**Flow Dual-Alignment Fusion.** According to Flow Dual-Alignment Fusion (FDAF) [8], the fusion module can be represented as:

(1) $$\begin{array}{c} \Delta p_{0,1}=Conv(Concat\left(\left[t_{0},t_{1}\right]\right), \end{array}$$

(2) $$\begin{array}{c} x=Concat(\left[t_{0}\left(p+\Delta p_{0}\right)-t_{1},t_{1}\left(p+\Delta p_{1}\right)-t_{0}\right]), \end{array}$$

where $t_{0}$ and $t_{1}$ are two different input images, *p* represents the position information of $t_{0}$ and $t_{1}$, and the position offset Δp is obtained by the two-layer Conv.

**MIX-FFN.** According to MIX-FFN [8], the image is input into the MIX-FFN for change prediction using convolution layers. The module can be represented as:

(3) $$\begin{array}{c} x_{out}=MLP\left(GELU\left(Conv_{3\times 3}\left(MLP\left(x\right)\right)\right)\right)+x. \end{array}$$

**Temporal Fusion Attention Module.** According to the Temporal Fusion Attention Module (TFAM) [31], dual-temporal features $T_{1}$ and $T_{2}$ are first aggregated to produce the aggregated feature $T_{c}$. This feature is created by concatenating the global average and max pooling results of $T_{1}$ and $T_{2}$. Next, the dual-temporal channel weights, i.e, $T_{c1}$ and $T_{c2}$, are calculated by one-dimensional convolutions. The Softmax function is then used for normalization to obtain the normalized dual-time channel weights $T_{c1}^{\prime }$ and $T_{c2}^{\prime }$.

(4) $$\begin{array}{c} T_{c}=Cat\left(Avg_{c}\left(T_{1}\right),Max_{c}\left(T_{1}\right),Avg_{c}\left(T_{2}\right),Max_{c}\left(T_{2}\right)\right), \end{array}$$

(5) $$\begin{array}{c} T_{c1},T_{c2}=Conv_{1}\left(T_{c}\right),Conv_{2}\left(T_{c}\right), \end{array}$$

(6) $$\begin{array}{c} T_{c1}^{\prime },T_{c2}^{\prime }=\frac{e^{T_{c1}}}{e^{T_{c1}}+e^{T_{c2}}},\frac{e^{T_{c2}}}{e^{T_{c1}}+e^{T_{c1}}}. \end{array}$$

The dual spatiotemporal weights $T_{s1}^{\prime }$ and $T_{s2}^{\prime }$ are calculated in the same way. Finally, the above parameters will generate output X through the following operation:

(7) $$\begin{array}{c} X=\left(T_{c1}^{\prime }+T_{s1}^{\prime }\right)T_{1}+\left(T_{c2}^{\prime }+T_{s2}^{\prime }\right)T_{2}. \end{array}$$

### 3.2. Overall Architecture

**Figure 2:**
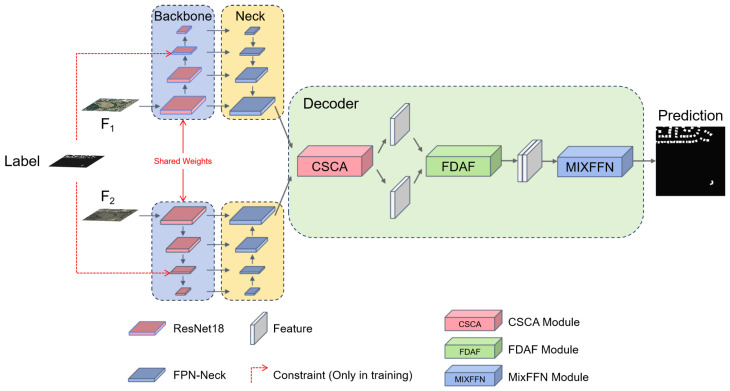
Overall framework of CINet. The blue section represents the backbone, the yellow section denotes the FPN-Neck, and the green section corresponds to the decoder.

The overall architecture of CINet is described in Figure 2. The architecture utilizes dual-temporal images as input images, including the pre-changed image $F_{1}$ and the post-change image $F_{2}$. It employs the ResNet-18 to extract multi-scale features at four scale levels. Initially, the decoder used FPN-Neck [32] to perform multi-scale feature fusion on the two sets of inputs. The resulting two fused feature maps are subjected to feature interaction through the proposed CSCA module. The interacting images are then fed into the FDAF [8] registration module for alignment. Finally, the aligned images are input into the MIX-FFN [8] for change prediction using convolution layers.

### 3.3. Cross-Spatial-Channel Attention Module

**Figure 3:**
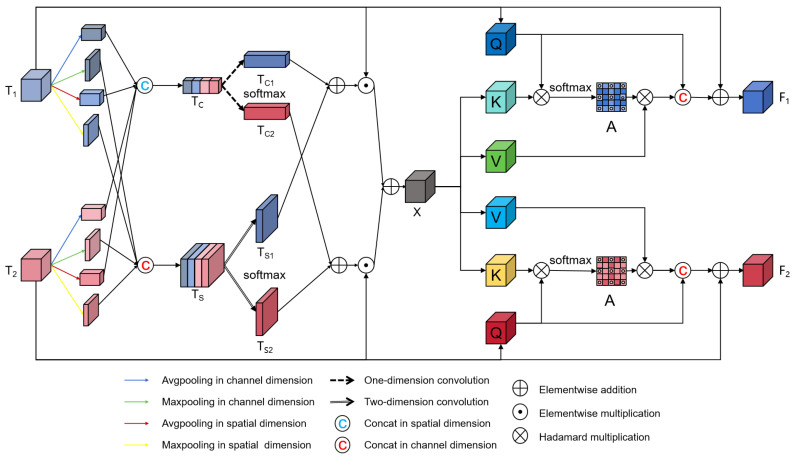
Structure of CSCA. $T_{1}$ and $T_{2}$ represent dual-temporal features. X represents the features that interact through the channel and spatial dimensions. $F_{1}$ and $F_{2}$ represent the results of cross-attention between the dual-temporal features and output.

To enhance the interaction of key regions in dual-temporal feature maps and reduce interference for stable performance improvement, we propose the CSCA module shown in Figure 3. Different from the existing interaction methods, we propose a mode of interaction with $T_{x}$, where $T_{x}$ is a new feature map that extracts valid information from input images $T_{1}$ and $T_{2}$. This pattern we call effective information exchange. The module interactively extracts important features from dual-temporal features to improve the feature recognition ability of the model in change detection. The input images $T_{1}$ and $T_{2}$ are generated by FPN-Net for multi-level input fusion. The CSCA module employs a Temporal Fusion Attention Module (TFAM) to identify key elements within the features. The TFAM combines channel attention and spatial attention to emphasize important areas in the features and utilizes temporal information to promote interaction between dual-temporal features. By ensuring that the total weight of the dual-temporal features sums to 1, the module retains relevant parts while discarding irrelevant information, effectively extracting features. The following formula represents the output of TFAM:

(8) $$\begin{array}{c} X=TFAM(T_{1},T_{2}), \end{array}$$

where X represents the important components extracted from the dual-temporal features. To better detect change pixels, the CSCA adopts a cross-attention mechanism that interacts separately with the dual-temporal feature maps, thereby obtaining important information from channel and spatial dimensions simultaneously. The input features $T_{1}$ and $T_{2}$ generate the queries $Q_{1}$ and $Q_{2}$, and the TFAM output X generates the keys $K_{x}$ and the values $V_{x}$, which are passed to the attention layer. By dot product $Q_{1}$ and $Q_{2}$ with $K_{X}$, respectively, the attention between input features and the effective information output of TFAM is obtained. Then, we multiply the attention matrix with the value vector $V_{x}$ to obtain the information after the interaction. The attention layer is represented as:

(9) $$\begin{array}{c} A\left(Q_{1,2};K_{x};V_{x}\right)=softmax\left(Q_{1,2}K_{x}^{T}\right)V_{x}. \end{array}$$

After obtaining the attention vectors, they are concatenated with the input features $T_{1,2}$ to generate new features $F_{1,2}$:

(10) $$\begin{array}{c} F_{1,2}=T_{1,2}+A\left(Q_{1,2};K_{x};V_{x}\right). \end{array}$$

By facilitating the interaction of important parts within the dual-temporal feature maps, the CSCA module enhances feature recognition capability and effectively reduces noise interference, significantly improving the accuracy of change detection.

### 3.4. Change Constraint

In remote sensing imagery, the changes in land cover typically involve small spatial areas, necessitating high spatial resolution to capture these changes adequately. Simultaneously, because of lots of instances containing remote sensing images, features with certain semantic information are required for accurate classification and identification. Typically, the early layers of the network are often focused on identifying basic elements in an image. These layers learn low-level features such as edges, corners, and textures through simple convolution operations. At a deeper level, the model captures abstract semantic features. Given that the different layers of the feature maps possess varying degrees of spatial resolution and semantic information, all these feature maps can provide useful information. However, due to the different characteristics of this information, it is not feasible to directly use them for change detection. Therefore, it is particularly crucial to introduce constraints in appropriate places to better adapt to the different characteristics of different information and the surface changes of different scales and to use lower computational cost and complexity in resource-limited environment.

The third-layer feature maps usually occupy a position between the low-level and high-level feature maps, exhibiting a certain level of abstraction while retaining relatively high spatial resolution. Consequently, they contain rich semantic information about the image content while preserving sufficient detail, making them well suited for RSCD. Compared to the deeper feature maps, the third-layer feature maps typically have lower computational complexity, which can somewhat reduce the model’s computational cost. These feature maps strike a balance between semantic information and spatial resolution, contributing to their effectiveness in change detection tasks. Therefore, the constraint mechanism can be designed so that the features obtained in the invariant region are more concentrated, while the features in the variable region are more dispersed. The goal is to maximize the use of useful information in the layer 3 feature map while suppressing unnecessary noise or variation and thus adaptability to the data distribution.

#### 3.4.1. Unchanged Area Constraint

The unchanged area refers to the region of land features that remain consistent between dual-temporal points, such as water bodies, roads, and buildings of two remote sensing images obtained at different times. These areas exhibit minimal visual alteration over time. Constraints on unchanged regions are typically implemented in the models by introducing pixel-level or region-level stability constraints. These constraints are based on the differences in pixel values, texture, or more advanced features to ensure that unchanged regions are correctly classified as non-change during change detection. Imposing constraints on unchanged regions helps reduce false alarm rates and enhance the precision of change detection. The formula for the unchanged region constraint is as follows:

(11) $$\begin{array}{c} X\_un=|f_{1}-f_{2}|\;*\;Mask, \end{array}$$

(12) $$\begin{array}{c} R\_un=\frac{sum(X\_un)}{sum(Mask)}, \end{array}$$

where $f_{1}$ and $f_{2}$ are dual-temporal features and Mask represents unchanged regions; then, in the selected unaltered region, the difference in the dual-temporal feature in the unchanged region gradually tends to zero through the constraint. Properly constraining the unchanged region helps reduce the false detection caused by the inherent properties of land features, thereby capturing the genuine change information more accurately.

#### 3.4.2. Changed Area Constraint

The change area refers to the region of land features that has undergone alterations between two distinct time points. These changes may encompass the construction of buildings, the growth of vegetation, the alterations in land use, and so forth. Typically, the constraints on change areas are established by accentuating the changes in land attributes or features. These features may include color, texture, shape, or even more advanced semantic information. By imposing restrictions on areas of change, the model can more effectively identify areas that need attention. Accurately capturing areas of change helps to accurately understand the location and potential impacts of geomorphic changes. Therefore, this paper proposes a method for constraints in change areas:

(13) $$\begin{array}{c} X\_ch=max((margin-|f_{1}-f_{2}|)*\left(1-Mask\right),0), \end{array}$$

(14) $$\begin{array}{c} R\_ch=\frac{sum(X\_ch)}{sum(1-Mask)}, \end{array}$$

where $f_{1}$ and $f_{2}$ are dual-temporal features and 1-Mask represents the change areas; the difference in dual-temporal features within the selected change regions should be ensured to be greater than a predefined margin. After that, normalization is carried out to improve algorithm detection capability. It helps to capture the change characteristics of the change area, improving the sensitivity and accuracy of change detection.

#### 3.4.3. All-Area Constraint

By simultaneously constraining the unchanged regions and change areas, the model can balance its focus on both the static and dynamic information during learning process and ensure the comprehensive constraints of dual-temporal images. This comprehensive constraint can better capture the differences between the images and reduce potential biases. Integrating the constraints from both the unchanged regions and the change areas encourages the model to learn which region remains constant across different times and which one undergoes the changes, facilitating effective information integration when generating the feature representations. This fully improves the accuracy and robustness of the model in identifying areas of change. By considering both unchanged and changed constraints, the model can better learn the feature representations of different regions in complex images. The formula for the all-area constraint is as follows:

(15) $$\begin{array}{c} R\_all=\frac{sum(X\_un+X\_ch)}{feat\_h*feat\_w}, \end{array}$$

where R_all represents the aggregated feature representation derived from the unchanged regions X_un and the change regions X_ch, normalized by the product of the height feat_h and width feat_w of the feature map. By fully considering the invariant constraints and change constraints, the model can understand the image content more deeply and detect the changes between images more effectively.

## 4. Experimental Results

Section 4.1 introduces the six datasets. Section 4.2 describes the parameter formulas used for change detection. The implementation details are described in Section 4.3. Related experimental results are elaborated in Section 4.4 and Section 4.5. Finally, a complete ablation experiment is presented in Section 4.6.

### 4.1. Dataset

**LEVIR-CD [5]** is a dataset that focuses on changes in building areas. It comes from an image provided by Google Earth. This includes 637 pairs of images with the resolution of 1024 × 1024. The dataset contains 31,333 building instances, marked with changed and unchanged places. We selected 445, 64, and 128 pairs of images for training, validation, and testing, respectively.

**S2Looking [2]** is a side-looking satellite image taken from the lowest point. It has 5000 pairs of satellite images containing 65,920 examples of new and demolished buildings in the countryside. The size of each image is 1024 × 1024. We selected 3500, 500, and 1000 pairs of images for training, validation, and testing, respectively.

**WHU-CD [33]** is a pair of images taken by aerial photography. It consists of two 32,507 × 15,354 images with 21,442,501 annotations in the conversion area. In the experimental phase, we are cropped into 256 × 256. We selected 4536, 504, and 2760 pairs of images for training, validation, and testing, respectively.

**SYSU-CD [34]** is a collection of aerial images of urban construction. It contains a large number of types of changes that can occur in the process of urban construction, including new buildings, plant coverage, and road expansion. It includes 20,000 pairs of images, each measuring 256 × 256 pixels. We used 12,000 pairs of aerial imagery as a training set and validated 4000 pairs, and the remaining 4000 pairs were used in the testing phase.

**HRCUS-CD [35]** contains changes in cities, old buildings, and farmland from 2010 to 2022. It split the two satellite images into 11,388 pairs of 256 × 256 images, including approximately 12,000 instances of changes. We divided these image pairs into 7974 training sets, 2276 validation sets, and 1138 test sets for experiments.

**DSIFN-CD [36]** is artificially collected from Google Earth. It captures changes in six Chinese cities, including housing construction, road facilities, and water. The six acquired pairs of images were split into 3988 pairs of images with 256 × 256 pixels. Among them, we selected 3600, 340, and 8 pairs of images for training, validation, and testing, respectively.

### 4.2. Evaluation Metrics

Our models are evaluated by the metrics (precision (*P*), recall (*R*), and *F*1 score) that are most commonly used in evaluating change detection models. Here is how they are calculated:

(16) $$\begin{array}{c} P=\frac{TP}{TP+FP} \end{array}$$

(17) $$\begin{array}{c} R=\frac{TP}{TP+FN} \end{array}$$

(18) $$\begin{array}{c} F1=\frac{2}{P^{-1}+R^{-1}} \end{array}$$

where *TP* is a true positive, *TN* is a true negative, *FP* is a false positive, and *FN* is a false negative. By comparing the size of these indicators, the performance of the model can be well demonstrated.

### 4.3. Implementation Detail

We use Open-CD [37], a PyTorch-based change detection toolkit, and related tools in open-mmlab [38] to build the model. When training, the cross-entropy (CE) loss function, AdamW optimizer, and cluster scheduling strategy are used. The weight decay and initial learning rate are set to be 0.05 and 0.005, respectively. The training was conducted on a single NVIDIA RTX A4000 GPU with a cluster size of eight. Models are trained on multiple datasets (LEVIR-CD, S2Looking, WHU-CD, SYSU-CD, HRCUS-CD, and DSIFN-CD). We also used various data augmentation techniques (resizing, cropping, rotating, and Gaussian blur) to improve the accuracy of the model. We evaluate the model’s accuracy by validating the data to select the best training weights and use them on a test dataset.

### 4.4. Main Results

In Table 1, Table 2, Table 3 and Table 4, we extensively evaluated our models on the six datasets mentioned above. The symbol ‘-’ indicates data that are not given in the paper in question. From these tables, it can be seen that the proposed CINet ranks first in F1 values obtained on all six datasets. On LEVIR-CD, the F1 value is as high as 92.00, which is 0.23 higher than the second highest value for ChangerEx. It is worth noting that on WHU-CD, the F1 value of our algorithm reached 90.61, even 0.72 higher than the second highest value for ChangerAlign. The F1 values of this algorithm on datasets such as S2Looking, SYSU-CD, HRCUS-CD, and DSIFN-CD are also higher than those of previous models. The proposed model performs well, mainly attributed to the use of the constraint module in the backbone, which effectively reduces the impact of noise and artifacts, and the adoption of the CSCA module in the decoder, which effectively improves the interaction between features. The experimental results fully demonstrate the superiority of the proposed constraint- and interaction-based change detection framework, and our model has more competitive performance than previous ones.

To further demonstrate the superiority of the proposed model, we present the statistical results of the P, R, and F1 metrics of our method on six datasets, as shown in Table 5. Generally speaking, it is difficult to obtain high precision and recall simultaneously. However, the P and R metrics of our algorithm performed well on all six datasets. Specifically, in the testing of the six datasets, the P indicator of our algorithm achieved first place once and second place twice, and the R indicator won first place three times and second place twice. It was worth noting that P and R simultaneously won first place once and the top two three times. This further proves the efficiency and robustness of the proposed model.

**Figure 4:**
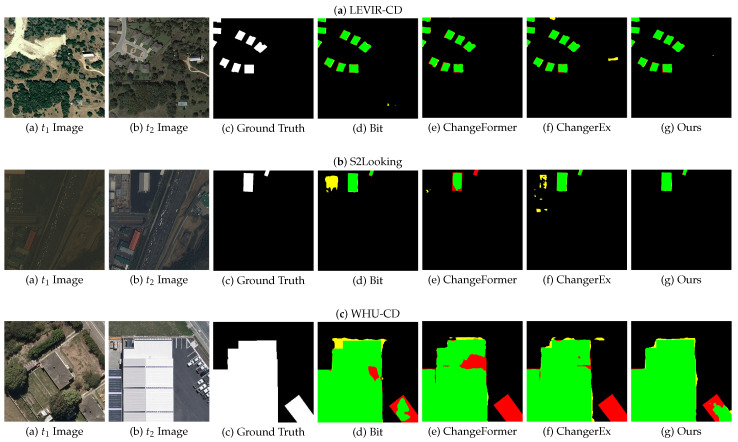
Visual comparison results of our model with other models on datasets, i.e, LEVIR-CD, S2Looking, and WHU-CD (from top to bottom). Green, yellow, and red represent TP, FP, and FN, respectively.

Figure 4 shows the comparison results between CINet and other methods on different datasets in the form of F1 visualization graphics. Since the datasets, i.e., LEVIR-CD, S2Looking, and WHU-CD, have high-resolution, diverse, and accurately annotated data representing different remote sensing platforms and scenarios, and the algorithms, i.e., Bit, ChangeFormer, and ChangerEx, represent advanced technologies in the field of RSCD covering different technical routes, the comparative experiment uses these three algorithms as comparison algorithms and conducts experiments on these three datasets. From Figure 4, we can see that compared with Bit, ChangeFormer, and ChangerEx, the proposed CINet model achieves the highest TP, FP, and FN. Meanwhile, CINet has better visual effects and its results are more in line with actual scene changes. The change regions and the boundaries extracted by CINet are more complete and accurate. This demonstrates the proposed constraint and interaction modules have advantages in change detection tasks, and CINet can effectively identify real changes, reducing false positives and false negatives.

### 4.5. Constraint Results

ResNet-18 (not pre-trained) is used as the backbone network in this paper. In ResNet-18, the network output is divided into key stages, and each stage generates a feature map with gradually decreasing spatial resolution. Specifically, we used four of these stages as output (resolutions of 1/4, 1/8, 1/16, and 1/32 of the original image). In order to enrich the representation capacity of the backbone, the performance improvement is investigated by using constraints in stages 1 to 4, respectively. The results indicate that for unchanged regions, implementing constraints in the third stage can achieve the most significant performance improvement, and there is also a good performance improvement in the second stage, as shown in Table 6. Similarly, when the constraints are applied to changed regions, the second stage makes the most significant progress, followed by the third stage, as shown in Table 7. To further determine which stage is more suitable for imposing comprehensive constraints, we applied constraints to both changed and unchanged regions, as shown in Table 8. We found the model performance was maximally improved when constraints were applied in the third stage. This result indicates that when the proposed constraints are applied to both unchanged and changed regions simultaneously, they can enhance the backbone network’s ability to extract change information from dual-temporal feature maps and improve the performance of the model.

### 4.6. Ablation Studies

Table 9 presents the results of ablation experiments for the proposed approach. Our baseline utilizes the ChangerAlign [8] module, which obtains an F1 score of 91.41. To better handle the scale variations, the multi-scale features from the backbone network are used to improve F1 scores (second row). Further performance enhancement is observed by incorporating the constraints on the third layer of the backbone (third row). It is worth noting that after adopting the CSCA module (fourth row), the performance of the model has been significantly improved, with an F1 score of 91.97. Other ablation experiment results can be found in lines five, six, and seven. Finally, we achieve an F1 score of 92.00 after applying all FPN-Neck, constraints and the CSCA, which fully verified the effectiveness of the proposed model.

## 5. Conclusions

This study proposes a novel approach for RSCD tasks, which achieves significant performance improvements by introducing constraint mechanisms in the model’s backbone and employing the CSCA interaction module. Experimental results on multiple remote sensing datasets demonstrate that CINet has excellent performance, which proves the effectiveness and feasibility of the constraint mechanism and the CSCA module. The approach introduced in this paper contributes to advancing RSCD technologies, providing valuable insights and directions for subsequent investigations in this field. The work in this paper introduces new perspectives and methodologies for research and applications in the remote sensing field, offering robust support for more precise identification and understanding of surface changes. Future endeavors may further explore the potential of constraint enhancement and the CSCA module to enhance the performance and efficiency of RSCD. 

## Figures and Tables

**Table 1:** Precision (%), recall (%), and F1 score (%) assessed on LEVIR-CD, S2Looking, and WHU-CD datasets. Red, blue, and **bold black** represent best, second-best, and third-best model results, respectively.

Method	Backbone	LEVIR-CD [5]			S2Looking [2]			WHU-CD [33]		
		Precision	Recall	F1	Precision	Recall	F1	Precision	Recall	F1
FC-EF [4]	-	86.91	80.17	83.40	81.36	8.95	7.65	77.24	68.88	72.82
FC-Siam-Conc [4]	-	91.99	76.77	83.69	68.27	18.52	13.54	76.94	69.74	73.17
FC-Siam-Diff [4]	-	89.53	83.31	86.31	83.29	15.76	13.19	71.61	73.40	72.49
DTCDSCN [39]	SE-Res34 [21]	88.53	86.83	87.67	68.58	49.16	57.27	90.15	89.35	**89.75**
STANet-Base [5]	ResNet18 [40]	79.20	89.10	83.90	25.75	56.29	35.34	-	-	-
STANet-BAM [5]	ResNet18 [40]	81.50	90.40	85.70	31.19	52.91	39.24	-	-	-
STANet-PAM [5]	ResNet18 [40]	83.81	91.00	87.26	38.75	56.49	45.97	**90.62**	86.26	88.38
CDNet [41]	ResNet18 [40]	91.60	86.50	89.00	67.48	54.93	60.56	89.80	83.30	86.4
BiT [7]	ResNet18 [40]	89.24	89.37	89.31	72.64	53.85	61.85	89.40	90.03	89.72
ChangeFormer [6]	MiT-b1 [17]	92.59	89.68	91.11	72.82	56.13	63.39	92.70	82.28	87.97
ChangerAlign [8]	ResNet18 [40]	93.30	89.59	**91.41**	71.62	**60.06**	**65.33**	90.20	**89.58**	89.89
ChangerEx [8]	ResNet18 [40]	**92.97**	**90.61**	91.77	**73.59**	60.15	66.20	89.71	88.94	89.32
Ours	ResNet18 [40]	93.07	90.96	92.00	71.78	61.86	66.45	91.46	89.77	90.61

**Table 2:** Precision (%), Recall (%), and F1 score (%) assessed on the SYSU-CD dataset. Red, blue, and **bold black** represent best, second-best, and third-best model results, respectively.

Method	SYSU-CD [34]		
	Precision	Recall	F1
FC-EF [4]	75.27	62.35	68.20
FC-Siam-Conc [4]	81.57	66.69	73.38
FC-Siam-Diff [4]	91.27	55.61	69.11
STANet-PAM [5]	77.60	78.10	77.80
HCGMNet [42]	84.31	75.07	**79.42**
HANet [43]	78.71	**76.14**	77.41
MSPSNet [44]	77.29	76.29	76.79
UPerNet [45]	81.13	74.38	77.61
BiT [7]	75.15	71.58	73.32
ChangeFormer [6]	70.84	59.40	64.62
RSP-BiT [7]	**86.29**	71.61	78.26
CGNet [46]	86.37	74.37	79.92
Ours	85.12	75.9	80.25

**Table 3:** Precision (%), Recall (%), and F1 score (%) assessed on the HRCUS-CD dataset. Red, blue, and **bold black** represent best, second-best, and third-best model results, respectively.

Method	HRCUS-CD [35]		
	Precision	Recall	F1
FC-EF [4]	**72.75**	50.30	59.48
FC-Siam-Conc [4]	53.95	66.95	59.75
FC-Siam-Diff [4]	64.29	67.76	65.98
U-Net [47]	60.87	69.75	65.01
SCDNet [48]	55.74	67.95	61.24
ChangeNet [49]	55.24	69.09	60.59
DSIFN [36]	59.42	74.76	66.21
DESSN [50]	62.18	**72.53**	**66.95**
BiT [7]	73.29	67.18	70.11
ChangeFormer [6]	67.68	64.45	66.55
Ours	78.47	78.55	70.47

**Table 4:** Precision (%), recall (%), and F1 score (%) assessed on the DSIFN-CD dataset. Red, blue, and **bold black** represent best, second-best, and third-best model results, respectively.

Method	DSIFN-CD [36]		
	Precision	Recall	F1
FC-EF [4]	61.80	57.75	59.71
FC-Siam-Conc [4]	59.08	62.80	**60.88**
FC-Siam-Diff [4]	68.44	58.27	62.95
STANet [5]	51.48	36.40	42.65
IFNet [51]	**63.75**	55.36	59.26
SNVNet [52]	64.15	57.09	60.41
BiT [7]	56.36	**62.79**	59.40
DASNet [53]	60.10	56.53	58.26
Ours	56.01	72.42	63.17

**Table 5:** Ranking statistics of P, R, and F1 indicators of the CINet on six datasets (unit: times), the numbers in parentheses indicate the ranking of a corresponding indicator. Red, blue, and **bold black** represent best, second-best, and third-best model results, respectively.

	P	R	(P, R)	F1
best	1	3	Top one: 1, (1, 1)	6
2nd best	2	2	Top two: 3, (1, 1) (1, 2), (2, 1)	0
**3rd best**	0	0	Top three: 3, (1, 1) (1, 2), (2, 1)	0
Others	3	1	3	0

**Table 6:** Experimental results by using the unchanged area constraint on LEVIR-CD. Red, blue, and **bold black** represent best, second-best, and third-best model results, respectively.

	Precision	Recall	F1
U-Constraint-1	93.73	89.48	91.56
U-Constraint-2	93.31	90.11	91.68
U-Constraint-3	93.59	89.89	91.70
U-Constraint-4	93.28	90.09	**91.65**

**Table 7:** Experimental results by using the changed area constraint on LEVIR-CD. Red, blue, and **bold black** represent best, second-best, and third-best model results, respectively.

	Precision	Recall	F1
C-Constraint-1	93.83	89.29	**91.50**
C-Constraint-2	93.31	89.95	91.60
C-Constraint-3	93.61	89.52	91.52
C-Constraint-4	93.38	89.68	91.49

**Table 8:** Experimental results by using the all area constraint on LEVIR-CD. Red, blue, and **bold black** represent best, second-best, and third-best model results, respectively.

	Precision	Recall	F1
A-Constraint-1	93.48	89.73	91.68
A-Constraint-2	93.43	89.96	**91.66**
A-Constraint-3	93.70	89.91	91.77
A-Constraint-4	93.48	89.86	91.63

**Table 9:** Ablation study of CINet on LEVIR-CD.

Baseline			Precision	Recall	F1
FPN-Neck	A-Constraint	CSCA			
			93.30	89.59	91.41
✓			93.37	90.27	91.61
	✓		93.70	89.91	91.77
		✓	93.41	90.58	91.97
✓	✓		93.37	90.27	91.79
✓		✓	93.08	90.93	91.99
	✓	✓	92.78	91.01	91.89
✓	✓	✓	93.07	90.96	**92.00**

## Data Availability

The data that support the findings of this study are available from Wei at this email address, wei_geng@nnnu.edu.cn, upon reasonable request.
